# Evaluating the Performance of Algorithms in Axillary Microwave Imaging towards Improved Breast Cancer Staging

**DOI:** 10.3390/s23031496

**Published:** 2023-01-29

**Authors:** Matilde Pato, Ricardo Eleutério, Raquel C. Conceição, Daniela M. Godinho

**Affiliations:** 1 Instituto de Biofísica e Engenharia Biomédica, Faculdade de Ciências, Universidade de Lisboa, 1749-016 Lisboa, Portugal; 2 Future Internet of Technologies-Lisbon School of Engineering (FIT-ISEL), R. Conselheiro Emídio Navarro 1, 1959-007 Lisboa, Portugal; 3 Lisbon School of Engineering (ISEL), R. Conselheiro Emídio Navarro 1, 1959-007 Lisboa, Portugal; 4 Physics Department, NOVA School of Science and Technology, NOVA University of Lisbon, 2829-516 Caparica, Portugal

**Keywords:** breast cancer staging, axillary lymph nodes, beamformer algorithms, microwave imaging

## Abstract

Breast cancer is the most common and the fifth deadliest cancer worldwide. In more advanced stages of cancer, cancer cells metastasize through lymphatic and blood vessels. Currently there is no satisfactory neoadjuvant (i.e., preoperative) diagnosis to assess whether cancer has spread to neighboring Axillary Lymph Nodes (ALN). This paper addresses the use of radar Microwave Imaging (MWI) to detect and determine whether ALNs have been metastasized, presenting an analysis of the performance of different artifact removal and beamformer algorithms in distinct anatomical scenarios. We assess distinct axillary region models and the effect of varying the shape of the skin, muscle and subcutaneous adipose tissue layers on single ALN detection. We also study multiple ALN detection and contrast between healthy and metastasized ALNs. We propose a new beamformer algorithm denominated Channel-Ranked Delay-Multiply-And-Sum (CR-DMAS), which allows the successful detection of ALNs in order to achieve better Signal-to-Clutter Ratio, e.g., with the muscle layer up to 3.07 dB, a Signal-to-Mean Ratio of up to 20.78 dB and a Location Error of 1.58 mm. In multiple target detection, CR-DMAS outperformed other well established beamformers used in the context of breast MWI. Overall, this work provides new insights into the performance of algorithms in axillary MWI.

## 1. Introduction

Breast cancer is the most frequent type of cancer in the world, accounting for approximately 2.26 million new cancer cases diagnosed in 2020 [1]. In most breast cancer cases during stages II to IV, cancer cells metastasize through lymphatic and blood vessels [2,3].

Current clinical practice assesses surgically removed Axillary Lymph Nodes (ALN)s as part of the TNM breast cancer staging (T relates to the tumor size, N relates to the number of lymph nodes that have been metastasized and M relates to whether the tumor has spread to other parts of the body) [4]. This comprises identifying the sentinel lymph node, which is the first axillary node in the lymphatic system to receive lymph drained from the primary breast tumor. The axillary nodes form a chain from the underarm to the collarbone. Surgeons classify them into three levels, according to their relationship to the pectoralis muscle on the chest: level I nodes are located in the lower part of the underarm, near the upper outer quadrant of the breast; level II between the medial and lateral border of the pectoralis muscle; and level III above the breast and below the center of the collarbone, along the inside border of the pectoralis muscle [4]. In most metastatic cases, the lymph follows in anatomical order, firstly reaching level I nodes, followed by level II and finally level III, in more advanced stages of breast cancer.

Neoadjuvant (i.e., preoperative) assessment of ALNs is of extreme importance to complete breast TNM staging. Current neoadjuvant non-invasive detection and diagnosis of ALNs using X-ray mammography, Ultrasound-Guided Biopsy, Magnetic Resonance Imaging, Positron Emission Tomography and Computed Tomography are often inconclusive [5,6,7]. The state-of-the-art procedure to evaluate axillary metastases is an intraoperative sentinel node biopsy which is a time-consuming surgical procedure limited to the first level nodes, which can result in the unnecessary removal of ALNs in upper levels [8,9]. This ultimately makes the patients’ physical recovery slow, and increases the risk of infection, lymphedema and paraesthesia [10,11]. Additionally, when ALN clearance is performed, patients lose the capability to drain lymph into the lymphatic system, requiring continuous monitoring and treatment, and hence preventing patients from a normal life and putting a financial burden on them and the healthcare systems [10,12]. Consequently, it is very important to keep improving the current techniques for diagnosis and therapy, as well as exploring new techniques that will ultimately improve the quality of life of patients diagnosed with breast cancer.

Microwave Imaging (MWI) has been studied in the last decades for early breast cancer [13] and stroke [14] detection. This technique is based on the fact that malignant tissues have different dielectric properties when compared to healthy tissues. Also, MWI presents several advantages compared to other imaging systems, namely the fact that it uses non-ionizing radiation, it is non-invasive, it is potentially low-cost and can be portable [13]. Recently, MWI has been studied for the early non-invasive detection of ALNs [15,16,17,18]. In most recent work [16,17,18], anatomically realistic models of the axillary region have been used to test these MWI systems.

Savazzi et al. [16] showed the challenges of detecting a single ALN in the presence of muscle in a 2D numerical study. Godinho et al. [17] presented an experimental evaluation with a single ALN in three positions showing the importance of finding adequate signal processing algorithms. Although promising results have been obtained, the level of complexity of their irregular shapes hampers the interpretation of the results. As observed in previous work, the adoption of proper artifact removal and beamforming algorithms is crucial to ensure satisfactory target detection. Several algorithms have been proposed in the last decades for medical radar MWI. Simple artifact removal algorithms such as Rotation Subtraction [19] and Average Subtraction [20] algorithms have been widely implemented. However, it has been reported that while they are computationally simple, their performance significantly decreases when the skin has varying thickness or the distance between the antenna and the skin varies, and it depends on the target location [21]. More sophisticated algorithms have been proposed, including filtered combination of signals [22,23], Singular Value Decomposition [24,25] or hybrid approaches [26], but their performance also depends on anatomical characteristics. Delay-And-Sum (DAS) and Delay-Multiply-And-Sum (DMAS) have been the reference beamforming algorithms for breast MWI. Several variations have been proposed but they usually increase the computational time of image reconstruction. One example is the iterative algorithm based on DAS proposed by Reimer et al. [27], which provided better results for breast target detection but increased the computational 10 fold.

In this paper, we explore axillary MWI, providing the following main contributions:

(1) performance analysis of MWI algorithms in simplified models, first presented in a preliminary study [15];

(2)study of the different anatomical characteristics in a controlled environment, adding incremental layers of complexity and compare different artifact removal and beamforming algorithms used in radar-based MWI;

(3) usage of well-established algorithms, which were still not properly evaluated for axillary imaging, and comparison of the imaging results for scenarios of varying complexity. The algorithms mentioned were proposed by Li et al. [20] and Bond et al. [22];

(4) proposal of a new beamforming algorithm to improve the image quality, called Channel-Ranked Delay-Multiply-And-Sum (CR-DMAS);

(5) presentation of additional insights about the challenges of axillary MWI for each layer of complexity and showing the importance of the chosen algorithms for multiple target detection.

The remainder of this paper is organized as follows: Section 2 examines the methodology regarding the simulation of an MWI system, signal processing techniques required to form an image and metrics to evaluate the imaging results. Section 3 summarizes the key results that reflect the performance of the selected image formation algorithms. Finally, Section 4 and Section 5 provide an overview of the work and address future directions.

## 2. Materials and Methods

**Figure 1:**
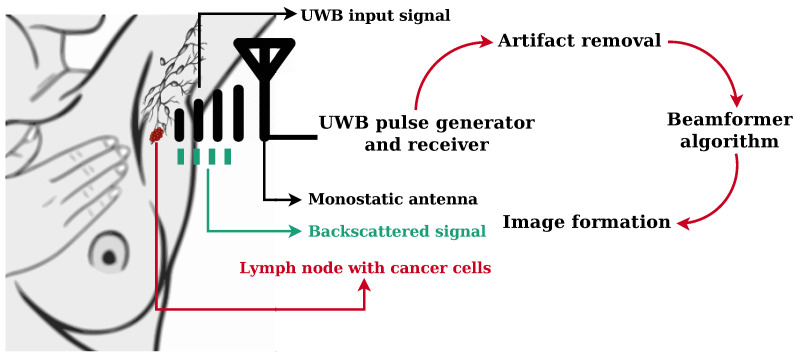
Diagram illustrating the proposed UWB Microwave Imaging radar system for the axilla.

In this study, we simulated an Ultra-Wide-Band (UWB) MWI radar system illuminating the axillary region with an ultra-short UWB pulse, emitted by UWB antennas equidistantly placed on the skin and recorded the backscattered signals at the same antennas (i.e., a monostatic system). An artifact removal algorithm was applied to the recorded signals to subtract the high backscattering response caused by the skin. Finally, a beamforming algorithm spatially focused the backscattered signals in order to create an energy profile of the axillary region to identify the presence and location of significant dielectric scatterers. High-energy regions potentially indicate the presence of ALNs. A diagram of the proposed UWB MWI radar system is shown in Figure 1.

### 2.1. Electromagnetic Simulation of an UWB Microwave Imaging Radar System

UWB microwave operates on the basis that electromagnetic scattering is generated by the contrast between tissues with different dielectric properties, particularly noticeable between normal and malignant tissues, mainly due to differences in water content. Numerical models of the body need to incorporate the different dielectric properties. The simulation of the propagation of electromagnetic waves in biological tissue is completed using the Finite-Difference Time-Domain (FDTD) method [28]. FDTD is based on a discrete solution of Maxwell’s time-dependent equations and has been used in many studies regarding the feasibility of breast MWI [29]. The frequency-dependency of the dielectric properties of biological tissue is implemented in the FDTD model with the following Debye formulation [29]:

(1) $$\varepsilon_{r}\left(\omega \right)=\varepsilon_{\infty }+\frac{\sigma_{s}}{j\omega \varepsilon_{0}}+\frac{\varepsilon_{s}-\varepsilon_{\infty }}{1+j\omega \tau }$$

where ω is the angular frequency, $\varepsilon_{\infty }$ is the permittivity at ω=∞, $\varepsilon_{s}$ and $\sigma_{s}$ are the static permittivity and conductivity at ω=0, respectively, and τ is the relaxation time constant, which is the time required for the displaced system, aligned with an electric field, to return to 1/e of its equilibrium value. The simulated input pulse is a 150 ps differentiated Gaussian pulse with a center frequency of 7.5 GHz and a −3 dB bandwidth of 9 GHz.

The Debye parameters used to model the dielectric properties of each type of tissue are shown in Table 1. Skin Debye parameters were obtained from published data in Zastrow et al. [30], healthy and malignant ALNs were modeled according to the dielectric properties found in studies by Cameron et al. [2], the dielectric properties for healthy tissue were based on the adipose tissue measurements described by Lazebnik et al. [31] and the muscle properties were based on those described by Gabriel et al. [32]. Although new insights about ALN average dielectric properties have been provided [33,34], in this paper we considered ALN heterogeneity within the models. A dielectric variation of 5% was randomly incorporated to 4-mm-side squares below the skin to mimic normal tissue heterogeneity.

Each model contained a 2-mm layer of skin and heterogeneous healthy tissue, which resulted in an axillary region depth of 100 mm. Different anatomical structures were added to the models to study their influence in ALN detection. A layer of subcutaneous adipose tissue below the skin can be included in the models, with a thickness varying with the Body Mass Index (BMI) according to Ludescher et al. [37]. Some models also included a 25-mm muscle layer which is 37-mm deep, parallel to the skin. ALNs were modeled with a bean-shaped curve [38]. In this paper, we only present models with ALNs whose longest axis is 12 mm. ALNs were placed at different depths of 20 mm and 37 mm under the skin (with reference to the center of the ALNs), respectively, within the range of ALN depths reported in [18]. An array of 16 antennas was placed on the skin, equally spaced and spread over a distance of 80 mm.

### 2.2. Microwave Imaging

A MWI, i.e., the energy profile, of the axillary region is possible by applying a skin artifact removal and beamformer algorithms to the recorded backscattered signals yielded from FDTD simulation.

#### 2.2.1. Skin Artifact Removal Algorithms

In our study, the recorded backscattered signals include responses from antenna reverberation, the skin surface, the skin-fat interface, the tissues that were modeled (for example muscle), as well as the signals from healthy and metastasized ALNs. The artifact produced by the skin is typically several orders of magnitude greater than the response from all other scatterers, and consequently its removal is essential so that other structures within the body can be observed. Several existing skin subtraction algorithms for MWI have been reported in the literature. We have implemented:

1. an algorithm proposed by Li et al. [20], which consists of creating a reference waveform by averaging all the stored waveforms and then subtracting the reference waveform from each of the original backscattered signals; and

2. an adaptive filtering algorithm proposed by Bond et al. [22] in which the artifact in each antenna is estimated as a filtered combination of the signal from all other antennas.

These two algorithms represent two fundamentals of artifact removal algorithms used in breast MWI and are used as a starting point to evaluate the performance in axillary imaging.

#### 2.2.2. Beamforming Algorithms

After the skin artifact was removed, a beamforming algorithm was used to focus the backscattered signals to each pixel in the output image. An efficient beamforming algorithm must identify the presence and location of dielectric scatterers, and provide high resolution, while suppressing clutter resulting from the normal heterogeneity of biological tissue. We implemented beamformers previously used in the context of breast MWI, such as (i) the Delay-And-Sum (DAS) [39], (ii) the Delay-Multiply-And-Sum (DMAS) [40], and (iii) the Channel-Ranked Delay-And-Sum (CR-DAS) [41]. These algorithms were chosen as they are widely used in the literature and are often used as a reference for evaluating the performance of algorithms.

**Figure 2:**
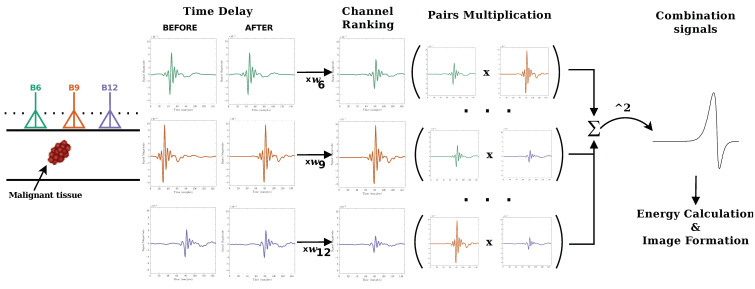
Diagram of the monostatic Channel-Ranked Delay-Multiply-And-Sum beamforming algorithm with M=16 antennas.

In this paper, we propose a new beamforming algorithm called Channel-Ranked Delay-Multiply-And-Sum (CR-DMAS). The CR-DMAS is inspired by the CR-DAS and the DMAS beamformers. Firstly, a time delay is added to the backscattered signal to account for the distance between each antenna and each synthetic focal point. Then, the signals are weighted according to their rank, following the process in [41], in which signals with shorter propagation distances are given extra weight. Signals are then multiplied in pairs, and finally all signals are summed and squared. The pairing multiplication procedure after the time shifting gives extra weight to channels with shorter propagation distances and allows additional clutter rejection. The schematic steps of the CR-DMAS algorithm is illustrated in Figure 2.

### 2.3. Performance Metrics

Beamformers performance and robustness were evaluated with performance metrics to:

(1) quantify the ability of the imaging system to successfully detect ALNs in the presence of other tissues (which introduce clutter);

(2) distinguish between malignant and healthy ALNs; and

(3) accurately locate the point of higher energy, which is assumed to correspond to the centre of the detected ALNs.

The following performance metrics were used:

(1) Signal-to-Clutter Ratio (SCR) [42,43], the ratio between the maximum response of the ALN and the maximum clutter response;

(2) Signal-to-Mean Ratio (SMR) [40,43], the ratio between the maximum response of the ALN and the average energy of all other tissues;

(3) Max-to-Max Ratio (MMR), which compares the maximum response of the ALN to the maximum response of the healthy ALN;

(4) Full Width Half Maximum (FWHM) [42,43], which measures the distance between the peak response of the detected ALN and the point at which the energy of its response drops to half, expressing the physical extent of the response of the ALN; and

(5) Localization Error (LE) [43], which reflects the distance between the peak response and the actual location of the ALN.

## 3. Results

In this section, we evaluate the performance of an MWI system detecting ALNs in the axillary region by processing backscattered signals acquired from several 2D FDTD axillary numerical models. For conciseness, only some of the implemented models will be presented.

### 3.1. Imaging One Lymph Node

Three different tests were completed considering models of heterogeneous healthy tissue with a single metastasized ALN. These will be discussed in the following subsections.

#### 3.1.1. Axillary Models with One ALN, Uniform or Sinusoidal Skin Layer

**Figure 3:**
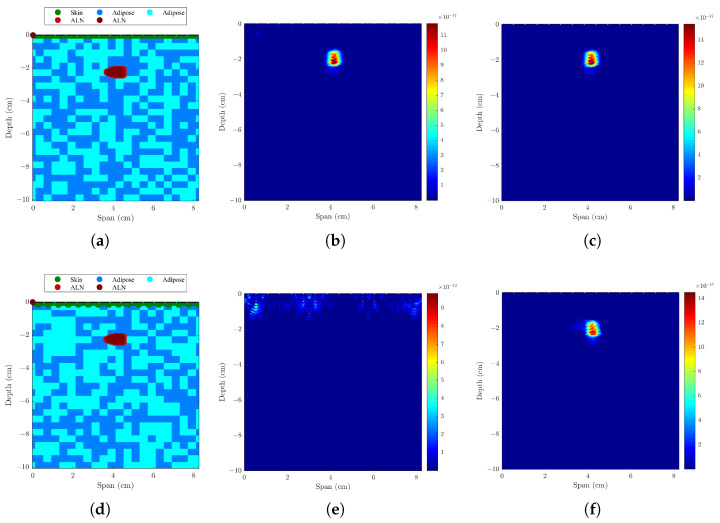
Models and the corresponding backscattered energy results from the implemented MWI system of an axillary region model with only one ALN and a (**a**–**c**) uniform skin layer or a (**d**–**f**) sinusoidal skin layer. The figure shows the simulated models in (**a**,textbfd), and the corresponding backscattered energy results using the CR-DMAS beamformer in (**b**,**e**), and (**c**,**f**) after applying Li’s and Bond’s artifact removal algorithm, respectively. The high intensity regions in (**b**,**c**,**e**,**f**) indicate the detection of the lymph node.

**Figure 4:**
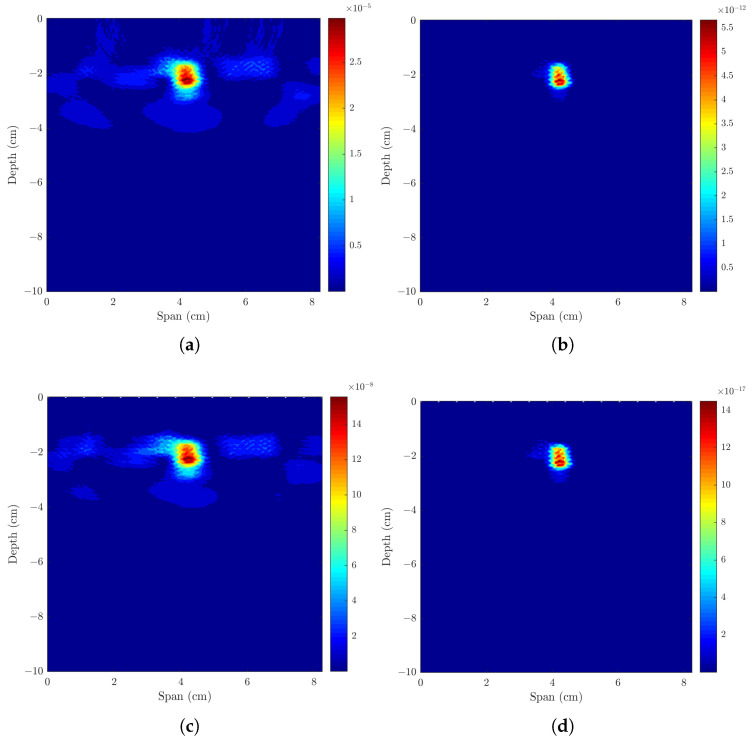
Backscattered energy results from the implemented MWI system of an axillary region model with a sinusoidal skin layer using four different beamformers—(**a**) DAS, (**b**) DMAS, (**c**) CR-DAS and (**d**) CR-DMAS—after applying Bond’s artifact removal algorithm. The high intensity regions indicate the detection of the lymph node.

The resulting backscattered images of models with a uniform and sinusoidal skin layer were compared, using different skin artifact removal algorithms and beamformers. The performance metrics are shown in Table 2 and Figure 3 shows imaging results with both Li’s [20] and Bond’s [22] skin artifact removal algorithms. When the skin is uniform in span width, both algorithms yielded good and similar results, detecting the metastasized lymph node with a minimum LE of 0.5 mm. However, when the skin is modeled with a sinusoidal shape, Li’s algorithm is not able to remove the skin response, whereas Bond’s algorithm is able to remove it, allowing a precise detection of the ALN with a LE lower than 1 mm. Bond’s algorithm is more robust, mainly due to the filter window that is optimized in the algorithm. Figure 4 shows the comparison between the four beamformers using Bond’s algorithm when a sinusoidal skin layer is present. Both DMAS and CR-DMAS provide imaging results with less clutter when compared to DAS and CR-DAS, respectively. The proposed algorithm in this paper—CR-DMAS—does not provide relevant improvements compared to DMAS in the tested conditions. The difference in SMR between DMAS and CR-DMAS is similar to the difference between DAS and CR-DAS. The LE remains unchanged no matter the type of beamformer used. FWHM values vary with the beamformers but are still within the size of the ALN. To simplify the interpretation of the components added in the following models, we considered a uniform skin layer. Also, since we showed Bond’s algorithm is more robust, only results from that algorithm are presented.

#### 3.1.2. Axillary Models with One ALN and Subcutaneous Adipose Tissue

**Figure 5:**
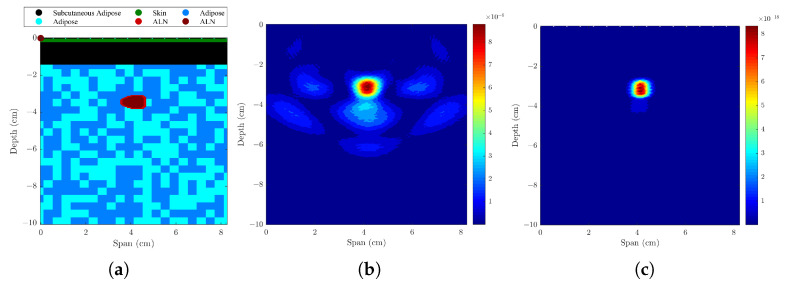
Model and the corresponding backscattered energy results from the implemented MWI system of an axillary region model with an ALN and a subcutaneous adipose layer. The figure shows (**a**) the model and the corresponding backscattered energy results using (**b**) DAS and (**c**) CR-DMAS beamformers after applying Bond’s artifact removal algorithm. The high intensity regions in (**b**,**c**) indicate the detection of the lymph node.

The results of adding a subcutaneous adipose tissue layer (12-mm layer for a normal weight woman [37]), which corresponds to a more realistic situation, are shown in the first section of Table 3 and in Figure 5. Results of SCR are very similar to when this layer is not included, and SMR decreased from 1.25 to 2.18 dB with DAS and CR-DAS algorithms, respectively. Both the LE and FWHM increased, but those values are still within the size of the lymph node, which suggests these changes are not relevant. These results demonstrate a deeper ALN is still identified correctly when only surrounded by adipose tissue.

#### 3.1.3. Axillary Models ALNs at Different Depths and Muscle Layer

**Figure 6:**
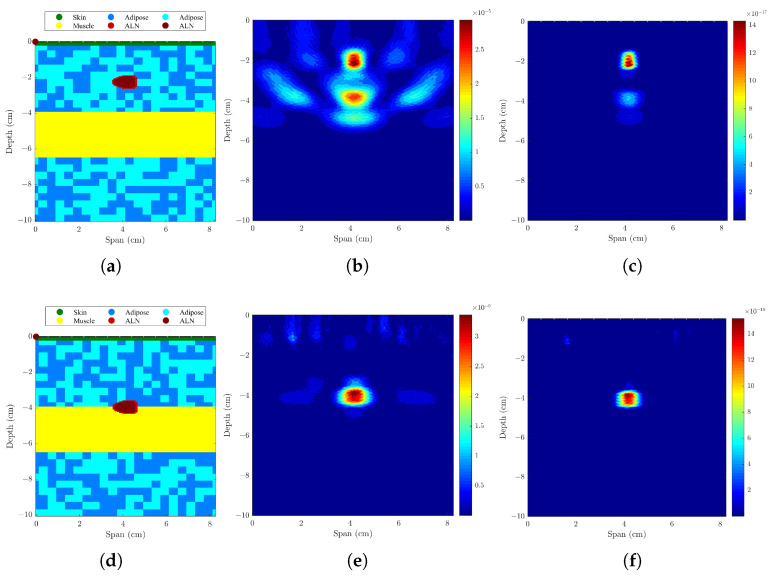
Models and corresponding backscattered energy results from the implemented MWI system of an axillary region model with a muscle layer and an ALN at different depths: (**a**–**c**) shallow ALN and (**d**–**f**) deep ALN. The figure shows the simulated models in (**a**,**d**), and the corresponding backscattered energy results using DAS and CR-DMAS beamformers in (**b**,**e**,**c**,**f**), respectively, after applying Bond’s artifact removal algorithm. The high intensity regions in (**b**,**c**,**e**,**f**) indicate the detection of the lymph node.

The effect of adding a muscle layer below the ALN was addressed in this test, considering ALNs at different depths. The results are shown in the last two sections of Table 3 and in Figure 6. The results show that in both situations, the ALN is detected in the correct position. In the case of shallowest ALN, there are some artifacts created by the muscle presence, and CR-DMAS performs better than the remaining beamformers, with higher SCR and SMR. When the ALN is deeper and placed on the surface of the muscle layer, Bond’s algorithm is capable of removing the skin and muscle response, resulting in an ALN detection with no artifacts.

### 3.2. Imaging Two Axillary Lymph Nodes

**Figure 7:**
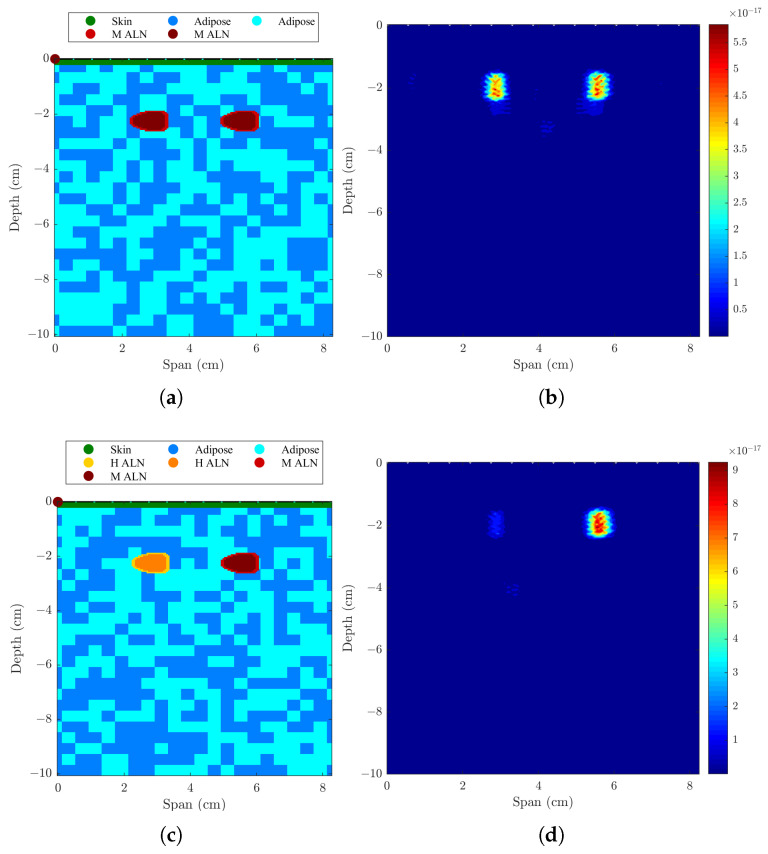
Models and corresponding backscattered energy results from the implemented MWI system of an axillary region model with (**a**,**b**) two metastasized (M ALN) ALNs, and (**c**,**d**) one healthy (H ALN) and one metastasized ALN, separated by 15 mm. The figure shows the simulated models in (**a**,**c**), and the corresponding backscattered energy results using the CR-DMAS beamformer after applying Bond’s artifact removal algorithm in (**b**,**d**). The high intensity regions in (**b**,**d**) indicate the detection of the lymph nodes.

The implemented skin artifact removal and beamforming algorithms were tested with other two numerical models of the axillary region, where two ALNs were considered simultaneously at a distance of 28 mm between their centers (i.e., 15 mm between their surfaces) and both at the same distance from the antenna-set limit. The performance metrics and resulting backscatered images are presented in Table 4 and Figure 7, respectively.

#### 3.2.1. Axillary Model with Two Metastasised ALNs

As shown in Table 4 and Figure 7a,b, when considering two metastasized ALNs separated by 28 mm, both ALNs are detected. All beamformers have similar detection rates as shown by MMR and DMAS with maximum value of 0.87 dB. Both the FWHM and LE are within the dimensions of the ALN.

**Figure 8:**
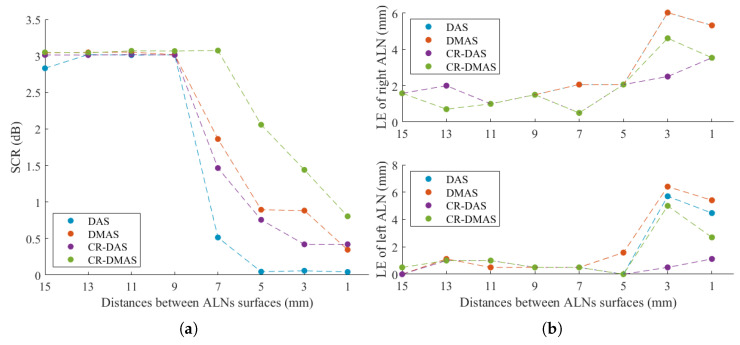
Plots of (**a**) Signal-to-Clutter Ratio (SCR) ratio and (**b**) Localization Error (LE) over distance between the surfaces of two metastasized ALNs.

**Figure 9:**
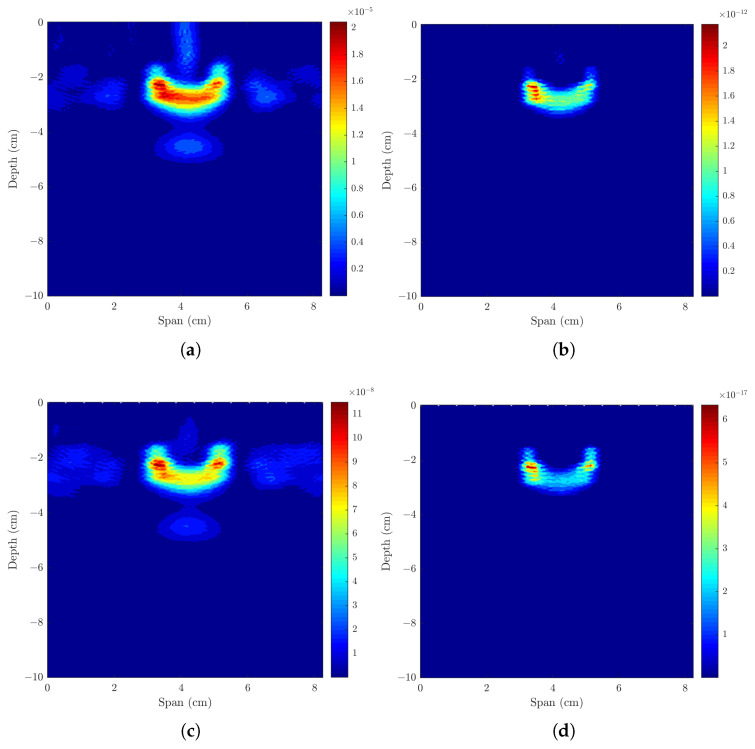
Backscattered energy results from the implemented MWI system of an axillary region model with two metastasized ALNs separated by 7 mm using four different beamformers—(**a**) DAS, (**b**) DMAS, (**c**) CR-DAS and (**d**) CR-DMAS—after applying Bond’s artifact removal algorithm. The high intensity regions indicate the detection of the lymph nodes.

To evaluate the resolution of the system, the distance between the surface of the ALNs was varied from 15 to 1 mm. Figure 8 shows the SCR and LE of both ALNs over the distances between different ALN surfaces. The system was able to differentiate between the two adjacent ALNs within the axilla for distances greater than 7 mm using all beamformers. CR-DMAS outperformed the remaining beamformers for distances equal to and lower than 7 mm, which corresponds to the distance when SCR started to decrease. Figure 9 shows the backscattered energy results using the four beamformers for two ALNs separated by a distance of 7 mm. Using CR-DMAS, 5 mm is the minimum value at which point metastasized ALNs are still distinguishable, which corresponds to the cross-resolution of this system. For shorter distances, SCR is too low and/or the LE of both ALNs is higher than half of their dimensions.

#### 3.2.2. Axillary Model with One Healthy and One Metastasized ALNs

The second part of Table 4 and Figure 7c,d shows the results when considering two ALNs with dielectric contrast separated by 28 mm. Both healthy and metastasized ALNs are detected at the correct location but with different detection contrasts, as expected. MMR shows there is higher contrast between the two ALNs when DMAS is used, followed by CR-DMAS.

## 4. Discussion

Axillary imaging yields a more complex imaging scenario when compared to breast or brain MWI, due to the limited angular view and the reduced size of ALNs compared to other anatomical structures. In this paper, we consider simplified scenarios and evaluate the performance of distinct skin artifact removal and beamforming algorithms when the complexity of the model increases. Anatomically realistic shapes of the axillary region have been considered in previous studies [16,18] but the study in our paper is essential to understand the effect of each component of the model in the quality of imaging results.

Moreover, considering the complexity of the axillary region shape, two main novelties were employed in this study: we considered a higher frequency band with a central frequency of 7.5 GHz when compared to a central frequency of 4 GHz in [16,18,44]; and we evaluated a planar antenna positioning spread over a distance of 80 mm when compared to a cylindrical antenna positioning [16,18]. A higher frequency band allows for reduced antenna dimensions and an increase in the density of antenna positions spread over the axillary region. This frequency range is sufficient to image shallower tissues, such as ALNs, and avoids the influence of the muscle response that may hamper ALN detection, as highlighted by Savazzi et al. [16].

We showed that in simple scenarios with uniform skin and with small proximity to other structures, ALN detection does not differ depending on the used beamforming algorithms. However, the proposed CR-DMAS algorithm yields better results than the remaining beamforming when two ALNs are close to each other, which may happen in a clinical scenario. Simple artifact removal algorithms, such as Li’s algorithm [20], do not yield satisfactory results for more complex scenarios. Bond’s algorithm [22] performed well for the tested cases but more sophisticated algorithms may be needed when increasing the complexity of the axillary region shape. These results highlight the importance of finding the proper algorithms for axillary imaging.

Another important contribution of this study is the evaluation of the cross-range resolution for two ALNs embedded in heterogeneous adipose tissue. As observed in Figure 8, when two ALNs are closer than 5 mm, for the considered configuration, the algorithms are not able to differentiate the two ALNs. To overcome this limitation, the number of antennas may be increased taking into account the available space and antenna dimensions.

## 5. Conclusions and Future Directions

We presented 2D models and corresponding electromagnetic simulation results aiming ALN detection and diagnosis using MWI technology. The results in this study complemented previous studies that show that MWI is a promising non-invasive technique that has the potential to identify the presence and location of ALNs. Our results showed ALNs detection with a maximum SCR around 3 dB and SMR around 24 dB, where the LE and FWHM were within the dimensions of the ALNs. Using adequate skin artifact removals and beamformers, the response of skin and muscles can be successfully removed from the imaging backscattered energy results providing an accurate detection of the ALN. We proposed a new algorithm based on previously established algorithms—CR-DMAS—which provides similar results to DMAS and outperforms both DAS and CR-DAS in most of the imaging results. Nonetheless, CR-DMAS shows its greatest advantage in multiple target detection when two ALNs are close to each other. The minimum distance between ALNs for an acceptable detection is 5 mm.

These results support the fact that MWI can be potentially used as a complementary diagnosis technique to breast examination, allowing a more complete diagnosis in terms of breast cancer TNM staging and consequently adequate follow-up treatment. This technique can potentially avoid health risks associated with the unnecessary removal of healthy ALNs which can prevent breast cancer patients from living a normal life.

Future work includes evaluating more complex scenarios, such as increasing the irregularity of the shape of the axillary region and the muscle. In those scenarios, the comparison of different antenna positioning and its impact on imaging results, as well as target detection resolution, is crucial. The interpretation of reconstructed images can also be aided by classification models as proposed by several authors for other applications [45,46]. These type of models handle microwave signals and provide more objective information regarding the presence of healthy or diseased body parts [47], or differentiation between benign or malignant tissues [48,49,50].

## Figures and Tables

**Table 1:** Dielectric properties for the tissues in the axillary region used in the Debye formulation [2,30,35,36].

Tissues		$\varepsilon_{\infty }$	$\sigma_{s}\left[S/m\right]$	$\varepsilon_{\infty }-\varepsilon_{0}$	τ[ps]
Skin		15.93	0.83	23.83	13
Adipose Tissue		3.12	0.05	1.59	
Muscle		21.66	0.89	33.24	
Healthy Lymph Nodes	Surface	3.60	0.19	1.71	
	Cross-Section	10.34	0.57	17.56	
Metastasized Lymph Nodes	Surface	6.86	0.67	12.37	
	Cross- Section	8.81	1.15	38.82	

**Table 2:** Performance metrics of all the beamformers using Li’s and Bond’s skin artifact removal algorithms for one ALN with uniform skin layer.

	Li’s Skin Artifact Removal				Bond’s Skin Artifact Removal			
Beamformer	SCR (dB)	SMR (dB)	LE (mm)	FWHM (mm)	SCR (dB)	SMR (dB)	LE (mm)	FWHM (mm)
ALN with uniform skin								
DAS	3.03	14.62	1.80	8.50	3.03	14.88	1.80	8.50
DMAS	3.03	24.05	0.50	6.50	3.02	24.38	0.50	6.50
CR-DAS	3.02	15.57	1.80	8.25	3.01	16.50	1.80	8.25
CR-DMAS	3.01	24.44	0.50	7.00	3.03	25.23	0.50	7.00
ALN with sinusoidal skin								
DAS	3.01	15.56	32.18	2.75	3.04	15.37	1.12	8.25
DMAS	3.04	19.76	32.18	1.75	3.04	24.25	1.12	6.00
CR-DAS	3.02	16.07	40.03	2.75	3.13	16.22	1.12	8.25
CR-DMAS	3.04	20.60	19.70	1.00	3.02	24.18	1.12	6.00

**Table 3:** Performance metrics of all the beamformers using Bond’s skin artifact removal algorithm for one ALN with new layers of subcutaneous adipose tissue or muscle.

Beamformer	SCR (dB)	SMR (dB)	LE (mm)	FWHM (mm)
ALN with subcutaneous adipose				
DAS	3.02	13.63	3.50	9.25
DMAS	3.07	22.73	3.50	7.50
CR-DAS	3.01	14.32	3.50	9.25
CR-DMAS	3.03	23.11	3.50	7.50
Shallowest ALN with muscle				
DAS	3.01	11.57	1.58	8.25
DMAS	3.01	19.00	1.58	6.50
CR-DAS	3.02	13.12	1.58	8.25
CR-DMAS	3.07	20.78	1.58	7.25
Deepest ALN with muscle				
DAS	3.02	16.68	0.71	9.00
DMAS	3.03	24.86	0.71	7.25
CR-DAS	2.54	15.34	0.71	9.00
CR-DMAS	3.02	23.24	0.71	7.25

**Table 4:** Performance metrics of all the beamformers using Bond’s skin artifact removal algorithms for two ALNs.

Beamformer	SCR (dB)	SMR (dB)	MMR (dB)	LE Left (mm)	LE Right (mm)	FWHM Left (mm)	FWHM Right (mm)
Two metastasized ALNs							
DAS	2.83	13.39	0.18	1.58	0.00	7.00	8.00
DMAS	3.04	20.70	0.87	1.58	0.00	5.00	5.50
CR-DAS	3.01	14.44	0.03	1.58	0.00	7.50	8.25
CR-DMAS	3.05	21.83	0.44	1.58	0.50	5.25	6.00
One healthy and one metastasized ALNs							
DAS	3.03	14.12	3.63	1.58	1.58	6.50	8.00
DMAS	3.01	23.17	8.37	1.12	1.58	4.50	6.75
CR-DAS	3.01	14.64	2.85	1.58	1.58	6.50	7.75
CR-DMAS	3.04	23.48	6.60	1.12	1.58	4.75	6.75

## Data Availability

Not applicable.

## Abbreviations

The following abbreviations are used in this manuscript:

ALNs	Axillary Lymph Nodes
CR-DAS	Channel-Ranked Delay-And-Sum
CR-DMAS	Channel-Ranked Delay-Multiply-And-Sum
DAS	Delay And Sum
DMAS	Delay-Multiply-And-Sum
FDTD	Finite-Difference Time-Domain
FWHM	Full Width Half Maximum
LE	Localization Error
MMR	Max-to-Max Ratio
MWI	Microwave Imaging
SCR	Signal-to-Clutter Ratio
SMR	Signal-to-Mean Ratio
UWB	Ultra-Wide-Band
